# Comparative assessment of invasive and noninvasive Methods for detection of total hemoglobin in gynecological patients' blood

**DOI:** 10.1186/cc11034

**Published:** 2012-03-20

**Authors:** AV Pyregov, SV Petrov

**Affiliations:** 1 Research Center for Obstetrics, Gynecology and Perinatology, Moscow, Russia

## Introduction

Safety of patients is possible to increase applying early detection of intraoperative and postoperative hemorrhage using the widening array of monitoring opportunities; not only the hemodynamic parameters, but the detection of total hemoglobin. Continuous noninvasive monitoring of total hemoglobin content is possible due to the Masimo Rainbow SET technology, using multiwave spectrophotometry.

## Methods

Seventy-eight patients aged 15 to 59 (35.9 ± 1.62) with laparoscopic gynecological operations were included in the research after permission of the ethics committee and signing the informing agreement. Total hemoglobin was detected by laboratory method invasively, discretely and delayed. Total hemoglobin was detected by another method oximetrically (SpHb) during the monitoring process on the platform Rainbow SET technology noninvasive, continuous, and promptly. SpHb was compared with total hemoglobin on the following stages of the research: before the operation, during the operation and in the early postoperative period. Statistical analysis was fulfilled by comparing real and tabular (critical) criteria of reliability - Student test.

## Results

During the detection of total hemoglobin by the laboratory method, the mean value was 121.5 ± 17.28 g/l, while oximetrically it occurred 118.6 ± 17.41 g/l. The real criterion of reliability (tr) was 0.85, the critical criterion of reliability (tcr) was 2.63.

## Conclusion

We did not discover statistically significant differences of total hemoglobin determined by two different methods. Thereby, noninvasive monitoring of total hemoglobin contention using multiwave spectrophotometry by Masimo Rainbow SET technology can serve as an appropriate replacement for the laboratory screening of hemoglobin.

